# Loss of Annexin A10 Expression Is Associated with Poor Prognosis in Early Gastric Cancer

**DOI:** 10.1267/ahc.20-00014

**Published:** 2020-09-24

**Authors:** Akira Ishikawa, Kazuya Kuraoka, Junichi Zaitsu, Akihisa Saito, Toshio Kuwai, Takahisa Suzuki, Hirotaka Tashiro, Kiyomi Taniyama, Wataru Yasui

**Affiliations:** 1 Department of Clinical Laboratory, National Hospital Organization, Kure Medical Center and Chugoku Cancer Center, 3–1 Aoyama, Kure 737–0023, Japan; 2 Department of Diagnostic Pathology, National Hospital Organization, Kure Medical Center and Chugoku Cancer Center, 3–1 Aoyama, Kure 737–0023, Japan; 3 Department of Gastroenterology, National Hospital Organization, Kure Medical Center and Chugoku Cancer Center, 3–1 Aoyama, Kure 737–0023, Japan; 4 Department of Surgery, National Hospital Organization, Kure Medical Center and Chugoku Cancer Center, 3–1 Aoyama, Kure 737–0023, Japan; 5 Honorary President, National Hospital Organization, Kure Medical Center and Chugoku Cancer Center, 3–1 Aoyama, Kure 737–0023, Japan; 6 Department of Molecular Pathology, Graduate School of Biomedical and Health Sciences, Hiroshima University, 1–2–3 Kasumi, Minami-ku, Hiroshima 734–8551, Japan

**Keywords:** annexin A10, early gastric cancer, prognostic biomarker

## Abstract

Gastric cancer (GC) is the third most common cause of cancer-related mortality. The diagnosis and treatment of early GC is a crucial strategy for prognostic improvement of GC. Annexin A10 (ANXA10), a calcium-/phospholipid-binding protein, is a member of the annexin family. The significance of ANXA10 expression in early GC remains unclear. This is the first report to investigate ANXA10 expression in early GC. We performed immunohistochemistry to evaluate ANXA10 expression in early GC, and the correlation between ANXA10 and clinicopathological factors. The loss of ANXA10 expression was detected in 63 (61.2%) of 103 early GC cases and significantly correlated with poor overall survival in patients. Sex, pT stage, pN stage, histology, and ANXA10 expression were associated with poor survival. Sex, histology, and ANXA10 expression were determined as independent predictors of survival in early GC patients. ANXA10 immunostaining could be a new decision-making biomarker in GC.

## I Introduction

Gastric cancer (GC) is the third most common cause of cancer-related mortality. Multiple genetic or epigenetic factors are thought to contribute to the carcinogenesis of GC [14]. Despite the tremendous progress achieved in GC research over several decades, the survival prognosis of advanced GC remains poor [18]. The diagnosis and treatment of early GC before it progresses to advanced GC is a crucial strategy for prognostic improvement of GC. Therefore, a prognostic biomarker that predicts the progression of early GC is required for clinical decision-making.

Annexin A10 (ANXA10), a calcium- and phospholipid-binding protein, is a member of the annexin family. Annexin family proteins have important roles in various cellular and physiological processes, including calcium ion channel formation, vesicle trafficking, exocytosis, endocytosis, and organization [2, 17]. ANXA10 expression is observed in stomach, duodenum, bladder, and kidney in normal organs [13]. In the stomach, ANXA10 is expressed in the nuclei of foveolar and oxyntic cells, while it is weakly expressed in the cytoplasm of intestinal metaplasia cells [5, 12].

ANXA10 expression is observed in approximately half of GC cases [13]. The importance of ANXA10 depends on Lauren classification, which is based on the structural morphology of GC [9]. Its expression is associated with a poorer prognosis in intestinal type GC. Alternatively, it is associated with a better prognosis in diffuse type GC. Some studies have reported that ANXA10 plays a tumor suppressive role by causing proliferation or apoptosis in GC [12]. According to these data, ANXA10 functions as a tumor suppressor for as long as the tumor cells express ANXA10. Previous studies, including ours, have examined advanced GC [5–7, 12]; however, the significance of ANXA10 expression in early GC has not been investigated.

In the present study, we aimed to examine the expression and prognostic tendency of ANXA10 in early GC using a public database. For that, we analyzed GC tissues from 103 early GC cases through immunohistochemistry (IHC), and analyzed the relationship between ANXA10 protein expression and clinicopathological characteristics. In addition, we examined the relationship between ANXA10 expression and prognosis of early GC cases.

## II Materials and Methods

### Tissue samples

Using a retrospective study design, 103 pT1b stage primary GC tissue samples were collected from patients diagnosed with GC, who underwent surgical or endoscopic resection between January 2010 and December 2014 at the Kure Medical Center and Chugoku Cancer Center (Hiroshima, Japan). Patients were monitored by their physicians until their death or the date of the last documented contact. For immunohistochemical analyses, the collected tissue samples were archival formalin-fixed and paraffin-embedded. The clinical characteristics of patients with early GC are summarized in Table 1. One representative tumor block from each patient was evaluated by IHC. Tumor stage was determined according to the TNM classification system. Histological classifications were determined based on the guidelines of the Japanese Research Society for Gastric Cancer. All patient samples were obtained with consent, and the present study was approved by the Ethical Committee for Human Genome Research of Kure Medical Center and Chugoku Cancer Center (2019-91).

### Immunohistochemistry

Immunohistochemical staining was performed according to several previous methods with some modifications including a western blotting study [5, 8]. Paraffin-embedded tissue blocks were available for immunohistochemical evaluation in 103 representative slides. The samples were cut into 4 μm-thick sections, deparaffinized, and rehydrated. Immunohistochemical staining was performed using a Ventana Benchmark ULTRA autostainer (Ventana Medical Systems, Tucson, AZ, USA) via the labeled streptavidin–biotin peroxidase method, and signals were visualized with 3,3'-diaminobenzidine. Antigen retrieval was performed using mild and standard cell conditioning 1 buffer. Endogenous peroxidase activity was not blocked. Sections were incubated with a rabbit monoclonal anti-ANXA10 antibody (1:500, Novus Biologicals, NBP1-90156) for 32 min. ANXA10 expression was scored in all tumors as positive or negative. The specificity of the antibody was confirmed previously [5]. The endogenous positive control was gastric epithelium on the same section, while the negative control was fibroblast. When >10% of tumor cells were stained, the immunostaining was considered positive for ANXA10. Using these definitions, two surgical pathologists (A.I. and K.K.), with no knowledge of the clinical and pathologic parameters or patient outcomes, independently reviewed the immunoreactivity of each specimen.

### Microarray dataset analysis

The GSE dataset (GSE55696) comprising 19 chronic gastritis and 19 early gastric cancer samples was downloaded from the GEO database (https://www.ncbi.nlm.nih.gov/geo/) and analyzed using GEO2R.

### Kaplan-Meier analysis

Kaplan-Meier analysis was performed for ANXA10 using the KMplot software from a database of public microarray data sets (http://kmplot.com/analysis). The results of 875 all stage GC patients and 67 stage I GC patients were collected from this database. To analyze the prognostic value of the probe, samples were split into two groups based on the cutoff value stipulated by the software program. Hazard ratios and *p* values (log-rank *p*) were indicated for each survival analysis.

### Statistical analysis

Correlations between clinicopathological parameters and ANXA10 expression were analyzed by Fisher’s exact test. Differences between survival curves were tested for statistical significance using log-rank test. Univariate and multivariate Cox regressions were used to evaluate the associations between clinical covariates and overall survival. The hazard ratio and 95% confidence intervals were estimated from Cox proportional hazard models. A *p* value less than 0.05 was considered statistically significant.

### Ethical approval and informed consent

All procedures followed were in accordance with the Ethical Committee for Human Genome Research of Kure Medical Center and Chugoku Cancer Center (2019-91) and the Helsinki Declaration of 1964 and later versions. Written informed consent or a substitute for it was obtained from all patients included in the study.

## III Results

### Loss of ANXA10 was identified and associated with poorer prognosis in GC

**Fig. 1.**
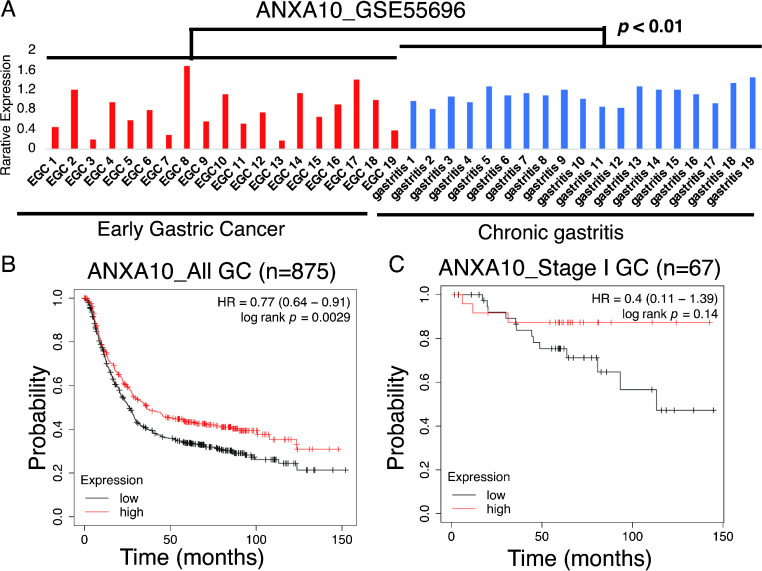
Annexin A10 (ANXA10) loss was identified in early gastric cancer (GC). (**A**) GEO2R analysis on 19 chronic gastritis and 19 early GC samples from GSE55696. (**B**) Survival probability of all GC patients with low or high ANXA10 expression. (**C**) Survival probability of stage GC patients with low or high ANXA10 expression.

We analyzed the level of ANXA10 in 19 chronic gastritis and 19 early GC samples in GSE55696 using GEO2R. The results suggested that the ANXA10 level was significantly lower in early GC samples than in chronic gastritis tissue samples (Fig. 1A). We also examined the relationship between ANXA10 expression and the prognosis of patients with early GC using a public database. We found that the all stage GC cases that demonstrated lower ANXA10 expression had worse prognosis (Fig. 1B). In addition, stage I GC cases with lower ANXA10 expression suggested a trend toward worse prognosis (Fig. 1C). Therefore, these results supported that low ANXA10 expression levels were associated with GC carcinogenesis and could lead to poor clinical outcomes in early GC patients.

### ANXA10 expression in early GC cells

**Fig. 2.**
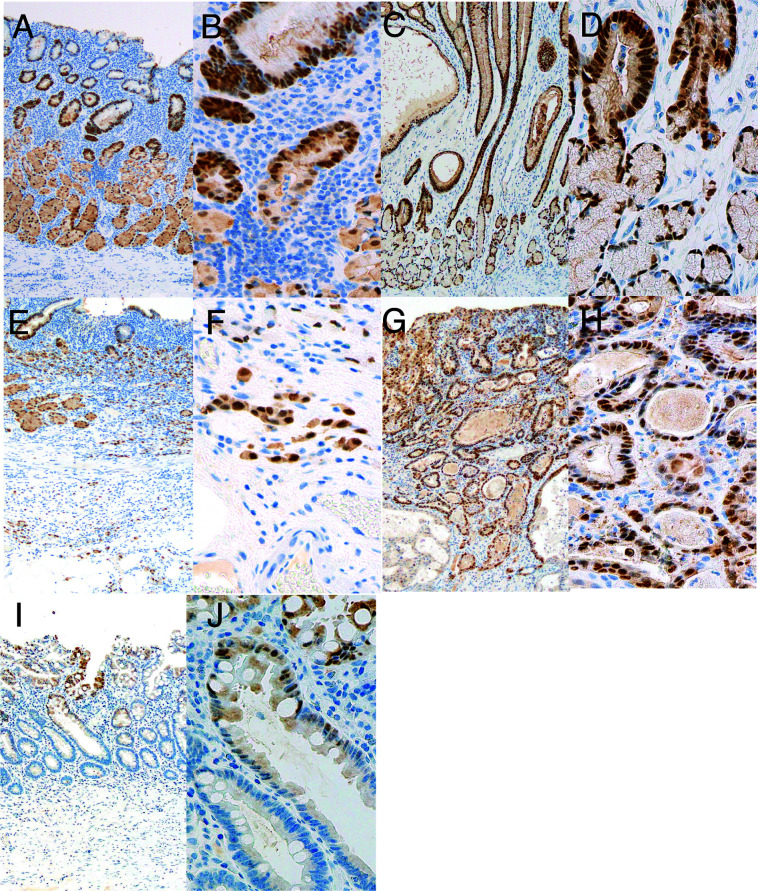
Representative images of annexin A10 (ANXA10) in non-neoplastic gastric mucosa and early gastric cancer (GC). (**A, B**) Immunohistochemical staining of ANXA10 in non-neoplastic gastric oxyntic mucosa. Original magnification: (**A**) 100× and (**B**) 400×. (**C, D**) Immunohistochemical staining of ANXA10 in non-neoplastic gastric pyloric mucosa. Original magnification: (**C**) 100× and (**D**) 400×. (**E, F**) Immunohistochemical staining of ANXA10 in diffuse type of early GC. Original magnification: (**E**) 100× and (**F**) 400×. (**G, H**) Immunohistochemical staining of ANXA10 in intestinal type of early GC. Original magnification: (**G**) 100× and (**H**) 400×. (**I, J**) Immunohistochemical staining of ANXA10 in intestinal metaplasia. Original magnification: (**I**) 100× and (**J**) 400×.

To analyze the correlation between ANXA10 expression and clinicopathological significance in early GC, we performed IHC of ANXA10 in 103 early GC tissue samples. We defined immunostaining as positive if 10% of the GC cancer cell nuclei showed any visible signals similar to previous studies that evaluated nuclear expression of ANXA10 in tumor cells [5]. In non-neoplastic mucosa, ANXA10 expression was observed in the nuclei of oxyntic cells (Fig. 2A, B) and pyloric gland cells (Fig. 2C, D) but not lymphoid cells, and was weakly positive in the cytoplasm of oxyntic cells (Fig. 2A–D). In early GC tissues, ANXA10 was detected in the nucleus and cytoplasm of tumor cells in both diffuse type GC (Fig. 2E, F) and intestinal type GC (Fig. 2G, H). In intestinal metaplasia, ANXA10 expression was variably observed in the nucleus or cytoplasm. A total of 63 (61.2%) early GC cases lost ANXA10 expression. Several GC cases demonstrated ANXA10 staining heterogeneity, with the percentage of ANXA10-stained GC cells in the range of 0 to 50%. No trend was observed for ANXA10 upregulation or downregulation at the invasive front.

### Correlation between ANXA10 expression and clinicopathological characteristics in early GC

Thereafter, we examined the relationship between ANXA10 staining and clinicopathologic characteristics (Table 2). A marginally significant difference was observed between ANXA10 expression and Lauren classification (*p* = 0.053, Fisher’s exact test). The results of Fisher’s exact test indicated no significant difference between ANXA10 expression and other clinicopathological factors, including age (*p* = 0.307), sex (*p* = 0.822), pT stage (*p* = 0.823), pN stage (*p* = 1.000), V (*p* = 1.000), and Ly (*p* = 0.314).

### Correlation between ANXA10 expression and survival of early GC patients

**Fig. 3.**
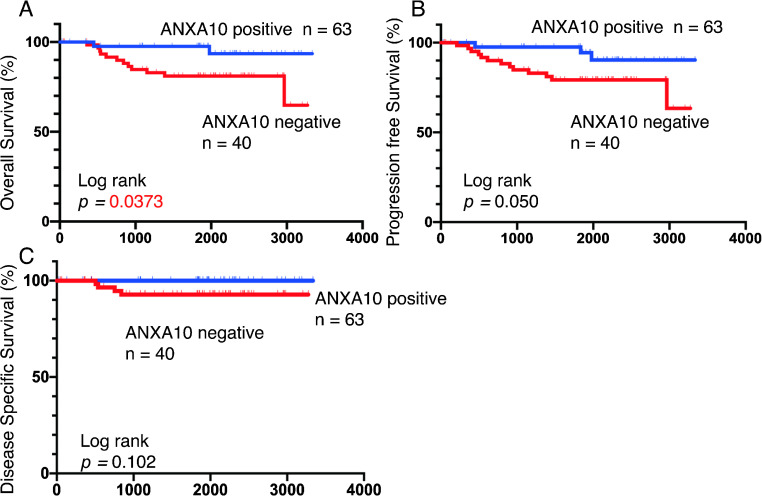
Kaplan-Meier curves of 103 annexin A10 (ANXA10)-positive and -negative early gastric cancer (GC) cases. (**A**) Overall survival. (**B**) Progression-free survival. (**C**) Disease-specific survival.

We subsequently examined the relationship between ANXA10 and survival of early GC patients. The 5-year overall survival rate was 97.6% for ANXA10-positive cases and 81.0% for ANXA10-negative cases. ANXA10-negative early GC cases had significantly poorer survival probability than those that were ANXA10-positive (*p* = 0.0373; Fig. 3A). A marginally significant difference was observed in the 5-year progression-free survival rate (Fig. 3B), and no significant difference was observed in the 5-year disease-specific survival rate (Fig. 3C). Thereafter, we performed univariate and multivariate Cox proportional hazard analyses to evaluate the potential use of ANXA10 expression as a prognostic classifier (Table 3). In univariate analysis, sex (HR, 2.344; 95% CI, 2.344–2.344; *p* < 0.01), pT stage (HR, 5.244; 95% CI, 1.044–95.21; *p* = 0.043), pN stage (HR, 3.768; 95% CI, 1.136–11.32; *p* = 0.032), histology (HR, 3.479; 95% CI, 1.196–10.72; *p* = 0.023), and ANXA10 expression (HR, 4.204; 95% CI, 1.144–27.03; *p* = 0.029) were associated with poor survival. In multivariate analysis, sex (HR, 2.037; 95% CI, 2.037–2.037; *p* < 0.01), histology (HR, 3.403; 95% CI, 1.085–11.90; *p* = 0.036), and ANXA10 expression (HR, 5.089; 95% CI, 1.355–33.06; *p* = 0.014) were found to be independent predictors of survival in early GC patients. These results suggested that ANXA10 is a potential biomarker for the identification of patients with poor prognosis.

## IV Discussion

Previously, we identified the significance of ANXA10 in advanced GC [5]. In the present study, we analyzed the expression and clinicopathological significance of ANXA10 in early GC, as the relationship between ANXA10 and carcinogenesis was unclear. To our knowledge, this is the first report to use such a large data series to determine the significance of ANXA10 expression in early GC cases.

Using data from a public database, we found that the loss of ANXA10 expression was associated with poor prognosis in early GC. A few previous reports have examined the mRNA expression level of ANXA10 in a small number of advanced GCs [6, 12]. In the public database, a significant difference was found in cases of all GCs; however, this was not so in the cases of early GCs. This may be due to the small number of GC cases in the database.

In immunohistochemical analysis, the loss of ANXA10 expression was observed in 61.2% of the 103 early GC cases. This result suggests that ANXA10 is downregulated during early carcinogenic processes of GC. Our study evaluated the ANXA10 expression in the nucleus; however, ANXA10 expression is also marginally positive in the cytoplasm. Some annexin family proteins have been found in the nucleus [2]. Previous reports have demonstrated that oxidative stress regulated the localization of annexins [15] and that annexin II is directly associated with the RNA of certain species [20]. This difference in cytoplasmic and nuclear localization may be meaningful and is one of the issues that warrant further studies in the future. To resolve this, next-generation sequencing could be used to examine the species of expressed ANXA10.

For comparison between ANXA10 expression and clinicopathological factors, a marginally significant difference was observed between ANXA10 expression and the histology status (*p* = 0.053), while no correlation was observed between ANXA10 and other clinicopathological factors. A limitation of this study is that the mechanism of ANXA10 remains unclear. Previously, it has been reported that reduced ANXA10 expression plays a role in GC progression through inhibition of cell proliferation and apoptosis [5–7]; however, no direct downstream pathways have been reported other than the insulin promoter factor 1 pathway [5]. ANXA11 plays an important role in regulating GC progression via the AKT/GSK-3b pathway [3]. There could be a similar downstream pathway for ANXA10 in GC. In neoplasms of other organs, especially colorectal cancer, ANXA10 is associated with CpG island methylation and BRAF mutation [1, 16]. A previous report showed that ANXA10, regulated by Cullin 4A, modulates invasion and metastasis of lung cancer [4]. It has been reported that ANXA10 expression is associated with poor prognoses in these carcinomas. Unlike in these neoplasms, ANXA10 expression confers a better overall prognosis in total GC, whilst the opposite effect among tumors is challenging in examining ANXA10.

In the present study, Kaplan-Meier analysis demonstrated that the loss of ANXA10 expression correlated with overall survival and served as an independent prognostic classifier of patients with early GC. To date, ANXA10 has been reported as an independent prognostic factor in hepatocellular carcinoma [10], ovarian cancer [21], cholangiocarcinoma [19], and thyroid cancer [11]. Despite various investigations, this is the first report of ANXA10 in early-stage gastric cancer. Overall, ANXA10 immunostaining is a clinically useful method to predict survival of early GC patients.

In conclusion, we demonstrated that ANXA10 expression is lost during the carcinogenic processes of GC, and found the clinical significance of ANXA10 in early GC. The expression status of ANXA10 was significantly associated with poor survival in early GC patients, and is an independent prognostic biomarker. ANXA10 immunostaining could be a new decision-making biomarker for early GC treatment.

## V Conflicts of interest

The authors declare that there are no conflicts of interest.

## VI Acknowledgments

The authors would like to thank the patients for allowing us to report their clinical information and data. We gratefully acknowledge the technical assistance of the following clinical technologists: Yasumura N, Kan A, Fujisawa H, Iwahiro K, and Kimura F.

## Figures and Tables

**Table 1.** Patients’ characteristics with early GC

All		103 (%)
Age (years)		
	Mean (range)	72.1 (44–93)
Gender		
	Male	76 (73.8)
	Female	27
Location		
	Upper third	13 (12.6)
	Middle third	54 (52.4)
	Lower third	36 (35.0)
pT stage		
	pT1b1	29 (28.2)
	pT1b2	74
pN stage		
	pN0	88
	pN1/2/3	15 (14.6)
Histological type		
	Well differentiated tubular (tub1)	34 (33.0)
	Moderately differentiated tubular (tub2)	24 (23.3)
	Poorly differentiated, solid (por1)	10 (9.71)
	Poorly differentiated, non-solid (por2)	21 (20.4)
	Papillary (pap)	11 (10.7)
	Signet (sig)	2 (1.94)
	Mucinous (muc)	1 (0.97)
Lauren classification		
	Intestinal-type	69 (67.0)
	Diffuse-type	34 (33.0)
Lymphatic invasion (Ly)		
	Ly0	73
	Ly1	30 (29.1)
Vascular invasion (V)		
	V0	88
	V1	15 (14.6)
ANXA10 expression		
	Positive	40
	Negative	63 (61.2)

**Table 2.** The relationship between the ANXA10 expression and clinicopathological characteristics in patients with ealry GC

		Annexin A10 expression		*p* value
		Positive (%)	Negative	
Age (years)				0.307
	<71	20 (46)	24	
	≥71	20 (34)	39	
Sex				0.822
	Male	29 (38)	47	
	Female	11 (41)	16	
pT stage				0.823
	pT1b1	12 (41)	17	
	pT1b2	28 (38)	46	
pN stage				1.000
	pN0	34 (39)	54	
	pN1/2/3	6 (40)	9	
V				1.000
	V0	34 (39)	54	
	V1	6 (40)	9	
Ly				0.314
	Ly0	30 (41)	43	
	Ly1	10 (33)	20	
Histology				0.053
	Intestinal-type	22 (32)	47	
	Diffuse-type	18 (53)	16	

**Table 3.** Univariate and multivariate Cox regression analyses of ANXA10 expression and survival of early GC patients

Characteristics		Univariate analysis			Multivariate analysis	
		HR (95% CI)	*p* value		HR (95% CI)	*p* value
Age						
	< 71	1 (ref.)	0.168			
	≥ 71	2.181 (0.729–7.958)				
Sex						
	Female	1 (ref.)	0.01<		1 (ref.)	0.01<
	Male	2.344 (2.344–2.344)			2.037 (2.037–2.037)	
pT stage						
	pT1b1	1 (ref.)	0.043		1 (ref.)	0.258
	pT1b2	5.244 (1.044–95.21)			3.140 (0.476–61.96)	
pN stage						
	pN0	1 (ref.)	0.032		1 (ref.)	0.206
	pN1/2/3	3.768 (1.136–11.32)			2.151 (0.636–6.678)	
Histology						
	Intestinal-type	1 (ref.)	0.023		1 (ref.)	0.036
	Diffuse-type	3.479 (1.196–10.72)			3.403 (1.085–11.90)	
ANXA10 expression						
	Positive	1 (ref.)	0.029		1 (ref.)	0.014
	Negative	4.204 (1.144–27.03)			5.089 (1.355–33.06)	
